# Sinonasal quality of life in patients after an endoscopic endonasal surgery of a sellar tumour

**DOI:** 10.1038/s41598-021-02747-5

**Published:** 2021-12-02

**Authors:** Vlastimil Novák, L. Hrabálek, J. Hoza, C.Hučko, D. Pohlodek, J. Macura

**Affiliations:** 1 Department of Neurosurgery, University Hospital Olomouc and Medical Faculty, Palacký University Olomouc, I. P. Pavlova 6, 779 00 Olomouc, Czech Republic; 2 Department of Ear, Nose and Throat Medicine, University Hospital Olomouc and Medical Faculty, Palacký University Olomouc, Olomouc, Czech Republic

**Keywords:** Surgical oncology, CNS cancer

## Abstract

Endoscopic endonasal approach uses the nasal cavity and paranasal sinuses to access the cranial base and may be a source of post-surgical morbidity in many patients with a sellar tumour. The objective of the presented study was to evaluate sinonasal quality of life and assess the effect of chosen reconstruction of the cranial base on the final condition. 65 patients, 33 male and 32 female who underwent an endoscopic endonasal surgery due to sellar expansion, were included into this prospective study. Sinonasal quality of life was evaluated using the Sinonasal Outcome Test-22 (SNOT-22) questionnaire before the surgery and six months after the surgery. Sinonasal quality of life was evaluated for the total cohort of patients and for patients after reconstruction (fascia lata, muscle) and without reconstruction. The minimum follow-up period was one year. There was no significant difference between the score (SNOT-22) before the surgery (average 14.4 points) and after the surgery (average 17.5 points), p = 0.067 in the whole cohort. Statistically significant differences were found in the following items—the need to blow nose, nasal congestion, loss of smell and taste, and thick discharge from the nose. The comparison of subgroups with and without the reconstruction yielded statistically significant differences in favour of patients with reconstruction in the following items—lack of high-quality sleep and feeling exhaustion. The endoscopic endonasal approach in patients with a sellar tumour is a gentle method with minimal effects on sinonasal quality of life over a period longer than six months. The most common complaints are the need to blow nose, nasal congestion, loss of smell and taste, and thick discharge from the nose. Cranial base reconstruction using the muscle and fascia lata seems to be a potential factor positively influencing sinonasal quality of life.

## Introduction

The endoscopic endonasal transsphenoidal approach is a surgical method suitable for most patients with a sellar tumour lesion. This surgical approach uses the nasal cavity and paranasal sinuses to access the cranial base and becomes the primary source of post-surgical morbidity in many patients^1^. These complications include sinusitis, synechiae, anosmia, epistaxis, septal perforation, crust formation, nasal congestion, and impaired mucociliary transport^1–4^. Although the transsphenoidal approach has been used in neurosurgery for more than 100 years, it has been only lately that endonasal morbidity became of interest. According to the PubMed database, after searching for the keywords „quality of life after transsphenoidal approach “ the first study with the focus on quality of life after transphenoidal apprach was published in 1998, with the following study pubslihed in 2011. We have found 43 studies with the principle focus in quality of life after transsphenoidal approach.

The objective of the presented study was to evaluate sinonasal quality of life after endoscopic endonasal transsphenoidal surgeries and assess the effect of chosen reconstruction of the cranial base on the final condition.

## Material and methods

This prospective study was performed from 1 January 2015 to 30 September 2019 and included 65 patients, 33 male and 32 female, who underwent a surgery using an endoscopic endonasal technique due to sellar expansion at the Department of Neurosurgery, Olomouc University Hospital. Inclusion criteria for the study were the age over 18 years, diagnosed sellar expansion indicated for an endoscopic transnasal transsphenoidal surgery, completion of the Sinonasal Quality of Life Questionnaire (SNOT-22) before the surgery and six months after the surgery. Patients with the history of an endonasal neurosurgery or otorhinolaryngology procedure, with a scheduled extended endoscopic approach, procedures using a nasoseptal flap, after previous radiotherapy of the sellar or sinonasal region were not included in the study. The minimum follow-up period was one year.

All patients underwent neurological, otorhinolaryngological, endocrinological and ophthalmological examinations before the surgery, including computer perimeter testing. The morphological diagnosis was based on contrast-enhanced MRI (Magnetic Resonance Imaging) scans of the pituitary gland, including navigation sequences. A CT (Computed Tomography) scan of the cranial base and paranasal sinuses was always performed before the surgery itself. Patients were informed about the benefits and risks of the surgery and signed the informed consent form to the surgical procedure.

### Surgical approach

The surgical procedure was performed using a binostril endoscopic technique based on collaboration with a neurosurgeon (L.H. and V.N.) and an otorhinolaringologist (J.H. and C.H.). The surgical procedure was performed in a supine position with the head fixed in a three-point clamp using neuronavigation. The nasal cavity was anemised (Sanorin^®^ 0.1%) and an endoscope with 0° or 30° optics (Storz^®^, Germany) was inserted into the nasal cavity, followed by lateralisation of the middle nasal conchae. Afterwards, the posterior part of the septum was resected 1.5 cm wide. Maximal sparing of olfactory epithelium was attempted. Septal branch of sphenopalatine artery was sacrificed in most cases. Wide opening of the sphenoid sinus followed. A part of the vomer was removed with a drill to the level of the lower sphenodial sinus, followed by removal of the intrasphenoidal mucosa and septum. After the identification of important anatomical structures, standard opening of the base of the Sella turcica durotomy and extirpation of the tumour were performed.

Reconstruction of the base of the Sella turcica was performed using the sandwich technique using the muscle, fascia lata, fat and tissue glue (Tisseel^®^) or no reconstruction was performed and the post-resection cavity was filled with oxycellulose (Surgicel^®^) and tissue adhesive (Tisseel^®^). Nasoseptal flap or middle turbinate graft was not used in any reconstruction in this study group. Nasal tamponade (Merocel^®^) was inserted into the nasal cavity at the end of the procedure and left there for two days. In case of peroperative cerebrospinal fluid leak, lumbar drainage was introduced for three to five days at the end of the surgical procedure.

Patients were advised to flush the nasal cavity with saline after the surgery and to avoid excessive increasing of intracranial pressure to prevent nasal leak of cerebrospinal fluid. All patients underwent a rhinological examination during the first week after the surgery and then, according to the local finding, were scheduled for regular check-ups until complete healing. Wash out of the nasal cavity (crust extraction), disruption of the synechiae, check-up of epithelialisation and purposeful search for possible leakage of cerebrospinal fluid were performed during the endoscopic examination. Pituitary MRI was routinely performed on the first post-surgical day, three months and one year after the surgery to evaluate radicality of the surgery. Peroperative or post-surgical complications have been recorded.

### Evaluation of sinonasal quality of life

A validated Czech version of the Sino Nasal Outcome Test 22 (SNOT-22) questionnaire was used to assess sinonasal quality of life^5^. This questionnaire contains 22 questions focusing on the symptoms and social/emotional consequences of nasal diseases. The rating scale uses the range of 0 to 5 points, with 0 points—no problem, 5 points—the most serious problem imaginable. The maximum total score is 110 points. Patients completed this questionnaire before the surgery and six months after the surgery. Sinonasal quality of life before and after the surgery was evaluated for the total cohort of patients and for patients after reconstruction and without reconstruction.

### Statistical analysis

Statistical processing of the cohort was performed using IBM SPSS Statistics 23 and all tests used the significance level p = 0.05. Ordinal data were described using medians, quartiles, minimum and maximum values, and mean values. Differences between dependent samples were statistically verified using the non-parametric Wilcoxon test. Differences between independent samples were statistically verified using the non-parametric Mann–Whitney U test.

Changes in the individual items were calculated as the difference between the values before and after the treatment to compare the subgroups of patients without cranial base reconstruction and with it (positive changes meant an increase—worsening of the parameter; negative changes meant a decrease—improvement of the parameter). Subgroups were compared based on changes of the individual items.

### Ethical approval

All procedures performed in studies involving human participants were approved by the ethical standards of the Ethics Committee of the University Hospital Olomouc (reference number: 151/21). All methods were carried out in accordance with relevant guidelines and regulations.

### Consent to participate

Informed consent was obtained from all individual participants included in the study.

## Results

The total cohort consisted of 65 patients, of which 33 were males and 32 females. The age range was 24–79 years (mean age 54.7 years). Table 1 summarizes the characteristics of the cohort—type of the tumour, individual complications, the need for prophylactic lumbar drainage. None of the patients in this study group experienced complications in need of another surgery.

**Table 1:** Characteristics of the cohort—gender, age, tumour type, prophylactic lumbar drainage, complications.

	Number of patients	
Total cohort	65	
Females	32	
Males	33	

Age	Range	Average
	24–79 years	54.7 years

Tumour type	Number of patients	
Afunctional macroadenoma	49 (75.4%)	
Acromegaly	4 (6.2%)	
Cushing’s disease	5 (7.7%)	
Acromegaly + prolactinoma	1 (1.5%)	
Prolactinoma	2 (3.1)	
Rathke’s cyst	2 (3.1%)	
Arachnoid cyst	2 (3.1%)	
Prophylactic lumbar drainage	12 (18.5%)	
Complications	12 (18.5)	
Diabetes insipidus (permanent)	4 (6.2%)	
Acute rhinosinusitis	6 (9.2%)	
Epistaxis	2 (3.1%)	
New visual deficit	0 (%)	
Focal neurological deficit	0 (%)	
Death	0 (%)	

There was no significant difference between the score before the surgery (average 14.4 points) and after the surgery (average 17.5 points), p = 0.067 in the whole cohort. When comparing individual items, statistically significant differences in terms of worsening were found in the following items—the need to blow nose, nasal congestion, loss of smell and taste, and thick discharge from the nose (Table 2).

**Table 2:** SNOT-22 scores before the surgery and 6 months after the surgery.

Item	Before the surgery (n = 65)				After the surgery (n = 65)				p
	Average	Median	Min	Max	Average	Median	Min	Max	
*1. Need to blow nose*	0.57	0	0	4	1.23	1	0	5	*0.0005*
2. Sneezing	0.57	0	0	4	0.77	0	0	3	0.163
3. Rhinitis (nasal discharge)	0.92	1	0	4	1.17	1	0	4	0.154
*4. Nasal congestion*	0.71	0	0	4	1.18	1	0	5	*0.013*
*5. Loss of smell and taste*	0.42	0	0	5	1.38	1	0	5	*0.00001*
6. Cough	0.54	0	0	5	0.49	0	0	4	0.572
7. Posterior (postnasal) rhinitis	0.32	0	0	4	0.58	0	0	4	0.054
*8. Thick discharge from the nose*	0.23	0	0	3	0.65	0	0	5	*0.004*
9. Pressure sensation (fullness) in the ears	0.29	0	0	3	0.43	0	0	3	0.14
10. Vertigo	0.49	0	0	4	0.43	0	0	3	0.775
11. Ear pain	0.18	0	0	4	0.34	0	0	4	0.094
12. Pain/pressure in the face	0.2	0	0	4	0.25	0	0	3	0.524
13. Difficult falling asleep	0.83	0	0	4	0.78	0	0	4	0.805
14. Waking up at night	1.03	1	0	4	1.2	1	0	5	0.445
15. Lack of high-quality night sleep	0.83	0	0	4	0.89	0	0	4	0.656
16. Fatigue after waking up	1.09	0	0	5	0.95	0	0	4	0.487
17. Feeling of exhaustion	1.26	0	0	5	1.14	1	0	4	0.516
18. Decreased productivity	1	0	0	5	1.02	1	0	4	0.858
19. Decreased concentration	0.94	0	0	4	0.83	0	0	4	0.543
20. Disgust/restlessness/irritation	0.86	0	0	4	0.83	0	0	5	0.804
21. Sadness	0.65	0	0	4	0.48	0	0	3	0.325
22. Embarrassment	0.51	0	0	4	0.43	0	0	4	0.596
General	14.4	8	0	73	17.5	13	0	58	0.067

Items with statistically significant change are marked in italics.

Table 3 reports a subgroup of patients with cranial base reconstruction. Statistically significant deterioration in terms of worsening was found in the following subgroups—the need to blow nose, nasal congestion, loss of smell and thick discharge from the nose. No statistically significant differences were recorded in the subgroup without reconstruction (Table 4).

**Table 3:** SNOT-22 scores before the surgery and six months after the surgery in a subgroup of patients after cranial base reconstruction.

Item	Before the surgery (n = 57)				After the surgery (n = 57)				p
	Average	Median	Min	Max	Average	Median	Min	Max	
*1. Need to blow nose*	0.63	0	0	4	1.3	1	0	4	*0.0008*
2. Sneezing	0.63	0	0	4	0.82	1	0	3	0.207
3. Rhinitis (nasal discharge)	0.95	1	0	4	1.23	1	0	4	0.147
*4. Nasal congestion*	0.7	0	0	4	1.14	1	0	5	*0.023*
*5. Loss of smell and taste*	0.46	0	0	5	1.39	1	0	5	*0.00003*
6. Cough	0.58	0	0	5	0.54	0	0	4	0.694
7. Posterior (postnasal) rhinitis	0.35	0	0	4	0.6	0	0	3	0.071
*8. Thick discharge from the nose*	0.26	0	0	3	0.74	0	0	5	*0.004*
9. Pressure sensation (fullness) in the ears	0.3	0	0	3	0.42	0	0	3	0.234
10. Vertigo	0.51	0	0	4	0.39	0	0	3	0.518
11. Ear pain	0.18	0	0	4	0.35	0	0	4	0.094
12. Pain/pressure in the face	0.23	0	0	4	0.28	0	0	3	0.524
13. Difficult falling asleep	0.81	0	0	4	0.77	0	0	4	0.805
14. Waking up at night	1.07	1	0	4	1.23	1	0	5	0.673
15. Lack of high-quality night sleep	0.91	0	0	4	0.86	0	0	4	0.733
16. Fatigue after waking up	1.18	0	0	5	0.96	0	0	4	0.219
17. Feeling of exhaustion	1.37	1	0	5	1.11	1	0	4	0.142
18. Decreased productivity	1.04	0	0	5	0.96	0	0	4	0.688
19. Decreased concentration	1	0	0	4	0.82	0	0	4	0.36
20. Disgust/restlessness/irritation	0.86	0	0	4	0.79	0	0	5	0.635
21. Sadness	0.65	0	0	4	0.4	0	0	3	0.139
22. Embarrassment	0.53	0	0	4	0.39	0	0	4	0.327
Generally	15.1	10	0	73	17.5	13	0	58	0.159

Items with statistically significant change are marked in italics.

**Table 4:** SNOT-22 scores before the surgery and six months after the surgery in a subgroup of patients without cranial base reconstruction.

Item	Before the surgery (n = 8)				After the surgery (n = 8)				p
	Average	Median	Min	Max	Average	Median	Min	Max	
1. Need to blow nose	0.13	0	0	1	0.75	0	0	5	0.317
2. Sneezing	0.13	0	0	1	0.38	0	0	3	0.655
3. Rhinitis (nasal discharge)	0.75	0	0	3	0.75	0	0	2	1
4. Nasal congestion	0.75	0	0	3	1.5	2	0	4	0.279
5. Loss of smell and taste	0.13	0	0	1	1.38	0	0	5	0.144
6. Cough	0.25	0	0	1	0.13	0	0	1	0.317
7. Posterior (postnasal) rhinitis	0.13	0	0	1	0.5	0	0	4	0.655
8. Thick discharge from the nose	0	0	0	0	0	0	0	0	1
9. Pressure sensation (fullness) in the ears	0.25	0	0	2	0.5	0	0	2	0.317
10. Vertigo	0.38	0	0	3	0.75	0	0	3	0.317
11. Ear pain	0.25	0	0	2	0.25	0	0	2	1
12. Pain/pressure in the face	0	0	0	0	0	0	0	0	1
13. Difficult falling asleep	1	0	0	4	0.88	1	0	3	1
14. Waking up at night	0.75	0	0	4	1	1	0	3	0.705
15. Lack of high-quality night sleep	0.25	0	0	1	1.13	1	0	3	0.102
16. Fatigue after waking up	0.5	0	0	3	0.88	0	0	3	0.705
17. Feeling of exhaustion	0.5	0	0	3	1.38	2	0	3	0.102
18. Decreased productivity	0.75	0	0	3	1.38	2	0	3	0.18
19. Decreased concentration	0.5	0	0	3	0.88	0	0	3	0.317
20. Disgust/restlessness/irritation	0.88	0	0	3	1.13	1	0	4	0.593
21. Sadness	0.63	0	0	3	1	0	0	3	0.564
22. Embarrassment	0.38	0	0	2	0.75	0	0	3	0.414
In total	8	7.5	0	24	17.3	16	0	33	0.176

The comparison of subgroups with and without the reconstruction yielded statistically significant differences in favour of patients with plastic surgery in the following items—lack of high-quality sleep and feeling exhaustion (Table 5).

**Table 5:** Comparison of subgroups of patients with and without cranial base reconstruction.

Change of the item	Reconstruction								p
	Yes (n = 57)				No (n = 8)				
	Average	Median	Min	Max	Average	Median	Min	Max	
1. Need to blow nose	0.67	0	− 3	4	0.63	0	0	5	0.414
2. Sneezing	0.19	0	− 3	3	0.25	0	− 1	3	0.722
3. Rhinitis (nasal discharge)	0.28	0	− 3	4	0	0	− 1	2	0.485
4. Nasal congestion	0.44	0	− 2	3	0.75	0	− 2	4	0.853
5. Loss of smell and taste	0.93	0	− 1	4	1.25	0	− 1	5	0.957
6. Cough	− 0.04	0	− 3	3	− 0.13	0	− 1	0	0.525
7. Posterior (postnasal) rhinitis	0.25	0	− 2	3	0.38	0	− 1	4	0.8
8. Thick discharge from the nose	0.47	0	− 1	3	0	0	0	0	0.386
9. Pressure sensation (fullness) in the ears	0.12	0	− 2	3	0.25	0	0	2	0.809
10. Vertigo	− 0.12	0	− 4	3	0.38	0	0	3	0.254
11. Ear pain	0.18	0	− 2	4	0	0	0	0	0.445
12. Pain/pressure in the face	0.05	0	− 3	3	0	0	0	0	0.75
13. Difficult falling asleep	− 0.04	0	− 4	4	− 0.13	0	− 4	2	0.636
14. Waking up at night	0.16	0	− 3	5	0.25	0	− 4	2	0.384
*15. Lack of high-quality night sleep*	− 0.05	0	− 4	4	0.88	0	0	3	*0.049*
16. Fatigue after waking up	− 0.21	0	− 4	4	0.38	0	− 3	2	0.147
*17. Feeling of exhaustion*	− 0.26	0	− 4	3	0.88	0	0	3	*0.033*
18. Decreased productivity	− 0.07	0	− 3	3	0.63	0	0	3	0.137
19. Decreased concentration	− 0.18	0	− 3	3	0.38	0	0	3	0.327
20. Disgust/ restlessness/ irritation	− 0.07	0	− 4	3	0.25	0	− 2	3	0.514
21. Sadness	− 0.25	0	− 4	3	0.38	0	− 3	3	0.279
22. Embarrassment	− 0.14	0	− 3	3	0.38	0	− 2	3	0.336

Calculated changes in the individual items are the differences of values (SNOT-22) measured before and after the treatment (positive changes denote an increase—deterioration of values; negative changes denote a decrease—improvement of values). Items with statistically significant change are marked in italics.

## Discussion

The transnasal endoscopic approach may adversely affect sinonasal function, specifically due to creation of wide sphenoidotomy and resection of the posterior part of the septum. Direct injury to ciliated epithelium leads to oedema, impaired mucociliary transport, crust formation and nasal congestion or increased discharge in the post-surgical period. Healing and clinical improvement usually occur within 3–6 months^1,6–8^. In the group presented in this study, there was no significant worsening in the total SNOT score—22 before surgery (14.4 points) and more than 6 months after surgery (17.5 points). Zimmer et al. prospectively evaluated sinonasal quality of life in patients after a pituitary adenoma surgery via the endoscopic transnasal approach using the SNOT—22 questionnaire. Their group consisted of 39 patients and the questionnaire was completed before the surgery, 1 month and 3 months after the surgery. The mean total score before the surgery was 23.4 points, 27.6 points 1 month after the surgery, and there was statistically significant improvement 3 months after the surgery, where the score was 16.2 points^9^. Little et al. performed a post-hoc analysis of sinonasal quality of life of a prospectively monitored multicentric cohort of patients after an endoscopic endonasal surgery of a pituitary tumour using the Nasal—12 score. The group consisted of 100 patients. The primary outcome showed statistically significant deterioration in sinonasal quality of life two weeks after surgery, with subsequent improvement to baseline levels within 3 to 6 months after the surgery. Negative factors influencing the outcome included the use of self-absorbing nasal tamponade, nasal splints, higher age, and female gender. On the contrary, application of fibrin glue to the sphenoid sinus led to lower crust formation and faster healing. The extent of pituitary tumour resection and the type of hormonal overproduction in functional adenomas exerted no effect on sinonasal quality of life^1^.

The comparison of individual symptoms in the presented study showed statistically significant worsening after the surgery in the following parameters: the need to blow nose, nasal congestion, loss of smell and taste and a thick discharge from the nose. In particular, olfactory dysfunction may be caused by a direct injury to olfactory epithelium or by obstruction of airflow to the olfactory mucosa. Netuka et al. published a prospective study evaluating the olfactory function of patients after an endoscopic endonasal surgery due to pituitary adenoma. Olfactory function was assessed using the Sniffin Stick test before the surgery, 3 months and 1 year after the surgery. The group consisted of 143 patients. Normosmia was demonstrated in 93.7% of patients before the surgery, in 95.8% 3 months after the surgery, and in 95.1% 1 year after the surgery. Hyposmia was demonstrated in 4.2% after the surgery, 2.1% 3 months after the surgery, and 1.4% 1 year after the surgery. Preoperative anosmia was demonstrated in 2.1%; in 2.1% 3 months after surgery and in 3.5% of cases 1 year after surgery^10^. Olfactory dysfunction develops more commonly in cases using a nasoseptal flap, generally in 14.4%^11^.

Statistically significantly better results were achieved in the following items—lack of high-quality sleep and feeling of exhaustion in the group of patients where cranial base reconstruction was performed using fascia lata, fat, muscle and tissue glue. We believe that reconstruction not only reduces the risk of post-surgical cerebrospinal fluid leak, bleeding, but also leads to quicker epithelialisation of the nasal mucosa. One of the disadvantages of this method is the requirement of graft harvest and pain at the incision site.

Limitations of this study include a shorter follow-up time, the absence of a control cohort and a smaller cohort of patients in the subgroup without reconstruction.

## Conclusion

Our study detected no significant difference in sinonasal quality of life before and 6 months after an endoscopic endonasal surgery due to sellar expansion during evaluation of the overall SNOT-22 score. The following complaints were statistically significantly more common after the surgery: the need to blow nose, the feeling of nasal congestion, loss of smell and taste, and thick discharge from the nose. Cranial base reconstruction using the muscle and fascia lata seems to be a potential factor positively influencing sinonasal quality of life.
